# Wavelet Fuzzy Brain Emotional Learning Control System Design for MIMO Uncertain Nonlinear Systems

**DOI:** 10.3389/fnins.2018.00918

**Published:** 2019-01-04

**Authors:** Jing Zhao, Chih-Min Lin, Fei Chao

**Affiliations:** ^1^School of Electrical Engineering and Automation, Xiamen University of Technology, Xiamen, China; ^2^Fujian Key Lab of Medical Instrument and Pharmaceutical Technology, Fuzhou University, Fuzhou, China; ^3^Department of Electrical Engineering and Innovation, Center for Biomedical and Healthcare Technology, Yuan Ze University, Taoyuan, Taiwan; ^4^Department of Cognitive Science, Xiamen University, Xiamen, China

**Keywords:** wavelet function, brain emotional neural network, fuzzy system, uncertainty, compensator controller

## Abstract

This paper aims to present a novel efficient scheme in order to more effectively control the multiple input and multiple output (MIMO) uncertain nonlinear systems. A wavelet fuzzy brain emotional learning controller (WFBELC) model is proposed, which is comprises the benefit of wavelet function, fuzzy theory and brain emotional neural network. When it is used as the main tracking controller for a MIMO uncertain nonlinear systems, the performances of the system, such as the approximation ability, the learning performance and the convergence rate, will be effectively improved. Meanwhile, the gradient descent method is used to adjust the parameters online of WFBELC and the Lyapunov function is employed to guarantee the rapid convergence of the control systems. For the sake of the further illustrating the superiority of this model, two examples of uncertain nonlinear systems, a Duffing-Holmes chaotic system and a Chua's chaotic circuit, are studied. After compared with other models, the test results show that the proposed model can be applied to obtain more satisfactory control performance and be more suitable to deal with the influence of the uncertainty of the MIMO nonlinear systems.

## Introduction

Uncertainty is an unavoidable problem in most technological cases. For the uncertain nonlinear systems, the acquisition of information is ordinarily limited and incomplete. Therefore, a model-free approach is usually used to effectively describe a system with the random characteristics in terms of structure and parameters (Lahmiri et al., 2013; Nikolić et al., 2013; Lin et al., 2014; Gosztolya and Szilagyi, 2015; Zhang et al., 2015). One of these effective methods is to combine the fuzzy inference system and neural network (NN) while building models. Then, the fuzzy neural network (FNN) not only offers a unique and flexible framework for knowledge representation but also processes the quick learning ability of NN. Moreover, wavelet analysis technology uses the dilation parameter and the translation parameter of mother wavelet, so the approximation of the signal can be more precise and more rapid due to the time-frequency localization properties of WF. When it is used as the activation function, it will possess the capability to analyse non-stationary signals to find the local details of the signal (Lin and Li, 2014). Therefore, combining the type-1 fuzzy inference, neural network (NN) and the wavelet function to construct wavelet fuzzy neural network (WFNN) will help to obtain more rapid global convergence and enrich the mapping relationship in a smaller number of iterations when dealing with the nonlinear and uncertain systems (Abiyev and Kaynak, 2008; Lu, 2011; Davanipoor et al., 2012; Liu et al., 2013).

Nevertheless, in the above neural networks the emotion factor is always ignored. In 1992, Le Doux found that the connection between a stimulus and its emotional consequences occurs in the amygdala of the brain (Le Doux, 1992). The brain has an amygdala and an orbital prefrontal cortex (Balkenius and MorÉn Jan, 2001). The amygdala system appears to be involved in excitatory emotional regulation, while the prefrontal system controls the response to changes in emotional emergencies (Rolls, 1986, 1995). Therefore, a mathematical model, brain emotional learning controller (BELC), has been established to describe the brain emotional learning (Sharbafi et al., 2010). The main structure of this model is divided into two parts. One is a sensory neural network that roughly corresponds to the amygdala, the other is an emotional neural network that roughly corresponds to the orbital prefrontal cortex. Self-learning and adjusting parameters are the main functions of the sensory neural network, and the functions of responding to external factors and establishing sensory-emotional correlation belong to the emotional neural network which has an indirect influence on the sensory neural network (Schultz et al., 1995). Moreover, they affect each other. In recent years, BELC has been widely applied for various different fields (Roshanaei et al., 2010; Dehkordi et al., 2011; Zarchi et al., 2011; Chung and Lin, 2015; Hsu et al., 2016; Zhou et al., 2017).

It is important to note that the conditioned reflexes occurring in the amygdala differ from those well-known in the cerebellum (Thompson, 1998; Yeo and Hesslow, 1998). It appears that conditioned reflex in the amygdala appears to establish emotional connections, while the cerebellum is involved in learning stimuli (Schultz et al., 1995). The emotional representation of a stimulus is independent of any response (Rolls, 1995). As for the same learning system, the amygdala and the cerebellum are different components (Gray, 1975). Therefore, the mathematical models and learning algorithms of the BELC differ from those of the cerebellum proposed by Albus (1975).

In this paper, we reconstruct a conventional BELC combined with a wavelet function and a fuzzy neural network. This novel model can be named as a wavelet fuzzy brain emotional learning controller (WFBELC). It takes advantages of a BELC, a wavelet function and a FNN to improve the learning ability over a conventional BELC. Finally, the effectiveness of the presented WFBELC is verified by some uncertain chaotic systems. The simulation and comparison results with the fuzzy Cerebellar Model Articulation Controller (FCMAC) and the BELC have shown that the proposed WFBELC can achieve much more favorable tracking performance. It can prevail over the forementioned control schemes when dealing with the influence of the uncertainty of the MIMO nonlinear systems.

As for the control of nonlinear systems, there are many control strategies (Zhong and Zhu, 2017; Chen et al., 2018; Fu et al., 2018; Zhong et al., 2018a,b; Zhu et al., 2018). Recently, sliding mode control (SMC) has attracted the interest of many researchers due to its powerful approach for nonlinear systems and incompletely modeled systems (Su et al., 2017). SMC also shows its high robustness by the capacity to cope with external disturbances (Wen et al., 2017). Based on these advantages, SMC has been applied in many applications. However, the main drawback of SMC is the chattering phenomenon, which has great influence on the trajectory tracking smoothness (Cui et al., 2017). To overcome this problem, a lot of studies have proposed some approaches (Joe et al., 2014; Zheng et al., 2014; Yu et al., 2017). The study in Joe et al. (2014) addressed that higher order sliding mode is an efficient approach to deal with the chattering. Therefore, in this study, the higher order sliding surface is used to enhance the control performance of the proposed algorithm, and also the robust compensator controller is used to cope the chattering and the residual error.

The remained of this paper is organized as follows. The modeling of wavelet fuzzy brain emotional learning controller is presented in section Modeling of Wavelet Fuzzy Brain Emotional Learning Controller. The updating algorithm and convergence analysis of WFBELC are presented in section Updating Algorithm and Convergence Analysis of WFBLC. The simulation results are provided in section Simulation Results. Finally, the conclusion is given in section Conclusions.

## Modeling of Wavelet Fuzzy Brain Emotional Learning Controller

### Fuzzy Inference Rules of WFBELC

As mentioned above, the brain has two parts: one is the amygdala responsible for the emotional judgment, and the other is the orbital prefrontal cortex responsible for the emotional control. So the fuzzy inference of the proposed WFBELC also consists of two type-1 fuzzy systems, i.e., the amygdala fuzzy system and the prefrontal fuzzy system.

The amygdala fuzzy system is defined as

(1) $$\begin{array}{l} \text{If }I_{1}\text{ is }s_{1}\text{ and }I_{2}\text{ is }s_{2},\ldots ,I_{n{\;}_{i}}\text{ is }s_{n{\;}_{i}}\;,\text{ then }u_{\;a\;o}=v_{i\;o}\\ \text{ for }i=1,2,\ldots ,n_{\;i},\;o=1,2,\ldots ,n_{\;o} \end{array}$$

**Figure 1:**
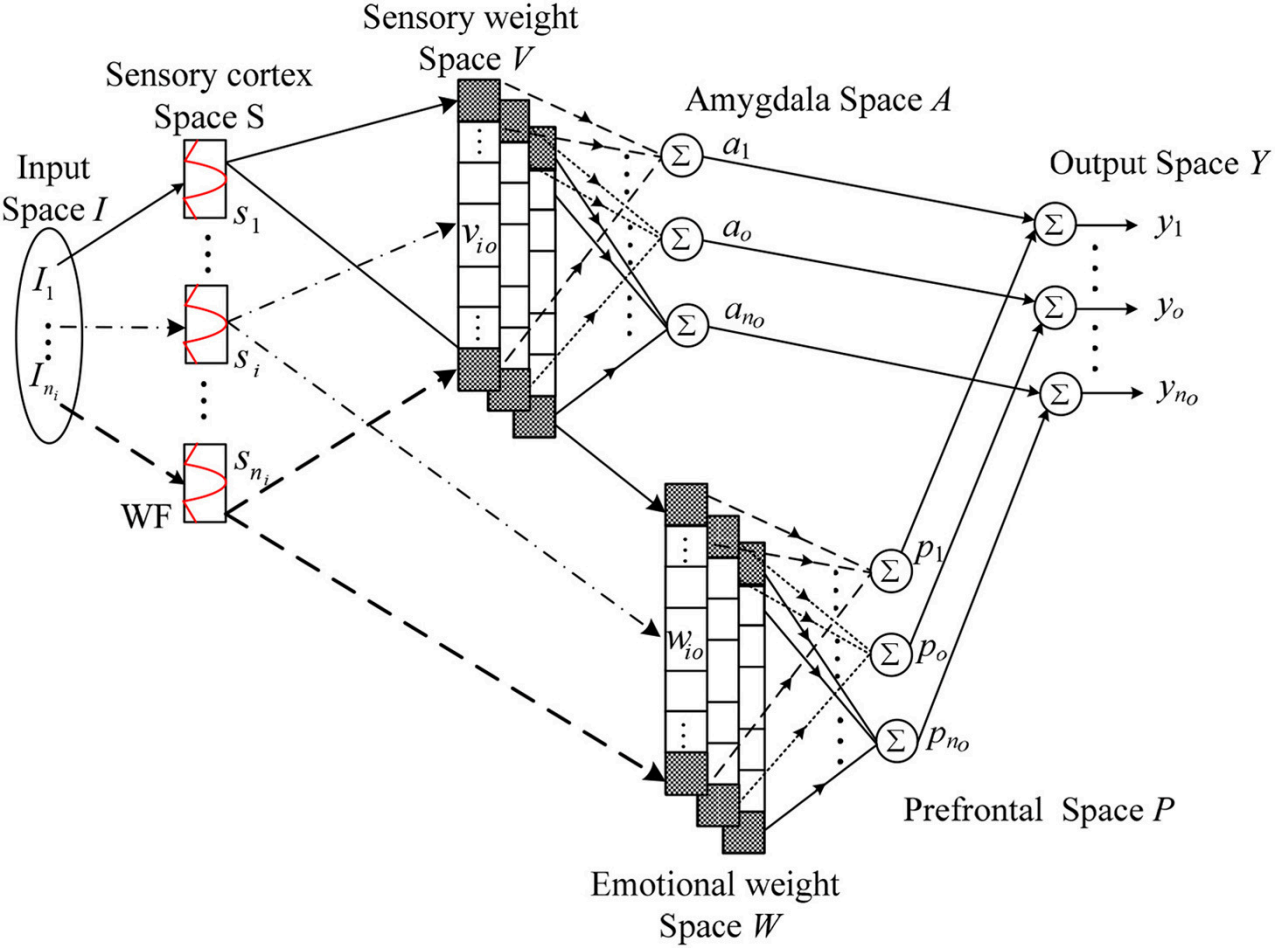
Structure of WFBELC.

The prefrontal fuzzy system is defined as

(2) $$\begin{array}{l} \text{If }I_{1}\text{ is }s_{1}\text{ and }I_{2}\text{ is }s_{2},\ldots ,I_{n{\;}_{i}}\text{ is }s_{n{\;}_{i}}\;,\text{ then }u_{\;p\;o}=w_{i\;o}\;\\ \text{ for }i=1,2,\ldots ,n_{\;i},\;o=1,2,\ldots ,n_{\;o} \end{array}$$

where *n*_*i*_ is the input dimension, *n*_*o*_ is the output dimension, *s*_*i*_ is the *i*-th input of the type-1 fuzzy set, *v*_*io*_ is the amygdala weight for the *o*-th output in the consequent part, *u*_*ao*_ is the *o*-th output of amygdala, *w*_*io*_ is the prefrontal weight for the *o*-th output in the consequent part, and *u*_*po*_ is the *o*-th output of prefrontal. The structure of this WFBELC is shown in Figure 1.

### Structure of WFBELC

The emotional system and sensory system receive inputs from sensory cortex. Both of them have 5 layers. The signal transmission and the main function of these layers are presented as follows.

1. **Layer 1**
  The layer 1 is the input space *I*. For a given, $I={\left[I_{1},\ldots ,I_{i},\ldots ,{\text{I}}_{ni}\right]}^{T}$ ∈ ℜ^*ni*^ *I*_*i*_ is an input state variable equaling to an actual input signal.
2. **Layer 2**
  The layer 2 is the sensory cortex space *S* which performs the fuzzification operation of WFBELC. In this space, wavelet functions are adopted as the basis function with the uniformly distributed translations and the same dilations in order to describe the linguistic terms.
  It has been proved that the integration of a Gaussian function is bounded and convergent. Therefore, all the derivatives of Gaussian function satisfy the admissibility condition of wavelet, and the n-order derivative of Gaussian function has the n-order vanishing moment. Thus, it is beneficial to compress data and eliminate noise, and possesses the better time-frequency localization properties (Zhao and Lin, in press). Therefore, in this paper, a type-1 Gaussian membership function is used as the mother wavelet. This Gaussian-type mother wavelet function can be expressed as:
  (3) $$\begin{array}{l} s_{i}\left(F_{i}\right)=-F_{i}\times \exp\left(\frac{-F_{i}^{2}}{2}\right) \end{array}$$
  Where *s*_*i*_ is the input from sensory cortex, which is the intensity of the individual stimulus components, *F*_*i*_ = (*I*_*i*_ – α_*i*_)/β_*i*_, where α_*i*_ and β_*i*_ are the *i*-th translation and the *i*-th dilation for the Gaussian-type wavelet of the *i*-th input *I*_*i*_, respectively.
  Because of the use of wavelet functions, the approximation ability for complex nonlinear functions is more effective than other basis functions, like the triangle basis function, the Gaussian basis function. Therefore, the learning speed is also increased.
3. **Layer 3**
  The layer 3 is the weight space *W*. In this space, each block is a fuzzy output, which indicates the inference part of the fuzzy rules.
  For the amygdala system, this space is known as the sensory weight space *V*, expressed in a vector form:
  (4) $$\begin{array}{l} {\underline{v}}_{o}={\left[v_{1o},v_{2o},\cdots ,v_{i\;o},\cdots ,v_{n{\;}_{i}\;o}\right]}^{T}\in {ℜ}^{n_{i}},\;\\ \;for\;o=1,2,\cdots ,n_{0} \end{array}$$
  For the prefrontal system, this space is known as the emotional weight space *W*, expressed in a vector form:
  (5) $$\begin{array}{l} {\underline{w}}_{o}={\left[w_{1o},w_{2o},\cdots ,w_{i\;o},\cdots ,w_{n{\;}_{i}\;o}\right]}^{T}\in {ℜ}^{n_{i}},\\ \;for\;o=1,2,\cdots ,n_{0} \end{array}$$
  where *v*_*io*_ and *w*_*io*_ represent the *o*-th weight value of the *i*-th input for the prefrontal system and the amygdala system, respectively.
4. **Layer 4**
  The layer 4 is the weighted sum of the sensory input *s*_*i*_, which can be solved by the method of center of gravity defuzzification.
  For the amygdala system, the defuzzification operator is defined as:
  (6) $$\begin{array}{l} a_{o}=\frac{\sum_{i}^{n\;i}s_{i}\times v_{i\;o}}{\sum_{i}^{n\;i}s_{i\;}}=\underline{\varphi }{\underline{v}}_{o} \end{array}$$
  For the prefrontal system, the defuzzification operator is defined as:
  (7) $$\begin{array}{l} p_{o}=\frac{\sum_{i}^{n\;i}s_{i}\times w_{i\;o}}{\sum_{i}^{n\;i}s_{i\;}}=\underline{\varphi }{\underline{w}}_{o} \end{array}$$
  where **φ** is in a vector form, and φ_*i*_ is the constant values of the *i*-th fuzzy rule. They can be defined as
  (8) $$\begin{array}{l} \varphi ={\left[\varphi_{1},\varphi_{2},....,\varphi_{ni}\right]}^{T}\in {ℜ}^{ni} \end{array}$$
  (9) $$\begin{array}{l} \varphi_{i\;}=\frac{s_{i\;}}{\sum_{i=1}^{n_{i}}s_{i\;}} \end{array}$$
  when $\overset{ni}{\underset{i}{\sum }}s_{i}=1$, (6 and 7) can be redefined as:
  (10) $$\begin{array}{l} a_{o}=\sum_{i}^{n\;i}s_{i}\times v_{i\;o} \end{array}$$
  (11) $$\begin{array}{l} p_{o}=\sum_{i}^{n\;i}s_{i}\times w_{i\;o} \end{array}$$
5. **Layer 5**
  The layer 5 is the output of the WFBELC. It is the result of interaction between the amygdala system and the prefrontal system. Thus, the *o*-th output of WFBELC is obtained as the following:
  (12) $$\begin{array}{l} y_{o}=a_{o}-p_{o},\;for\;o=1,2,\cdots ,n_{o} \end{array}$$

## Updating Algorithm and Convergence Analysis of WFBELC

### Updating Algorithm for the Brain Emotional Learning Controller

The emotional learning of the brain is achieved by updating the weights *v*_*io*_ and *w*_*io*_. According to the neurophysiological prototype, the main function of the amygdala is to predict and respond to specific emotional hints. By adjusting the orbital frontal cortex, the difference between amygdala output and emotional implication tends to minimized (Lucas et al., 2004). Therefore, in view of the emotional learning approach of the brain, the parameters adaptation laws of the amygdala system and prefrontal system are respectively applied as

(13) $$\begin{array}{l} \Delta v_{i\;o}=\eta_{v}\left[s_{i}\times \max\left(0,\theta_{o}-a_{o}\right)\right] \end{array}$$

(14) $$\begin{array}{l} \Delta w_{i\;o}=\eta_{w}\left[s_{i}\times \left(y_{o}-\theta_{o}\right)\right] \end{array}$$

where η_*v*_ is a learning-rate in the amygdale cortex and η_*w*_ is a learning rate in the prefrontal cortex.

The parameter θ_*o*_ is an adjustment denoting the emotional signal or reinforcing signal for the *o*-th output of WFBELC, which is a function of several parameters. In this paper, θ_*o*_ is represented as

(15) $$\begin{array}{l} \theta_{\;o}=(\sum_{i=1}^{n{\;}_{i}}\lambda_{i}\times I_{i})+(\gamma_{o}\times y_{o}) \end{array}$$

where λ_*i*_ and γ_*o*_ are the signal constant gains.

In order to represent the capability of forgetting the previous emotion signals, a maximum term is also added to Equation (13) as suggested in Fatourechi et al. (2001). Thus, the weight of amygdala cannot be decreased because the max function adjusts the weight monotonically. However, the prefrontal's learning rule is essentially different from the amygdala's. The orbitofrontal connection weight, seen from Equation (14), can resize the value to achieve the required output.

The updating laws for the weights of the amygdala system and the prefrontal system are written as:

(16) $$\begin{array}{l} v_{i\;o}\left(k+1\right)=v_{i\;o}\left(k\right)+\Delta v_{i\;o}\left(k\right) \end{array}$$

(17) $$\begin{array}{l} w_{i\;o}\left(k+1\right)=w_{i\;o}\left(k\right)+\Delta w_{i\;o}\left(k\right) \end{array}$$

### Gradient Descent Algorithm for the Sensory Cortex Space

To update the translation parameter *m*_*i*_ and the dilation parameter σ_*i*_ for a wavelet function, which are used in the sensory cortex space *S*, the normalized iterative gradient decent algorithm is applied. The back propagation is designed to deduce the parameter adaptation laws.

Firstly, an energy function *E* is defined as

(18) $$\begin{array}{l} E\left(k\right)=\frac{1}{2}\sum_{o=1}^{n_{o}}{\left(T_{o}\left(k\right)-y_{o}\left(k\right)\right)}^{2}=\frac{1}{2}\sum_{o=1}^{n_{o}}e_{o}^{2}\left(k\right) \end{array}$$

where *e*_*o*_(*k*) = *T*_*o*_(*k*) –*y*_*o*_(*k*) denotes the *o*-th error, *T*_*o*_(*k*) is the *o*-th target output, *y*_*o*_(*k*) is the *o*-th output of WFBELC.

Thus, the parameter updating learning law can be derived according to

(19) $$\begin{array}{l} \underline{\alpha }(k+1)=\underline{\alpha }(k)+\Delta \underline{\alpha }(k)=\underline{\alpha }(k)-\eta_{\alpha }\frac{\partial E}{\partial y_{o}}\frac{\partial y_{o}}{\partial \underline{\alpha }} \end{array}$$

(20) $$\begin{array}{l} \underline{\beta }(k+1)=\underline{\beta }(k)+\Delta \underline{\beta }(k)=\underline{\beta }(k)-\eta_{\beta }\frac{\partial E}{\partial y_{o}}\frac{\partial y_{o}}{\partial \underline{\beta }} \end{array}$$

where η_α_ is the learning rate of translation, η_β_ is the learning rate of dilation, and **α** = [α_1_, α_2_, …, α_*i*_, …, α_*ni*_]^*T*^, **β** = [β_1_, β_2_, …, β_*i*_, …, β_*ni*_]^*T*^

The gradient operation factors $\frac{\partial y_{o}}{\partial \underline{\alpha }}$ and $\frac{\partial y_{o}}{\partial \underline{\beta }}$ are defined as

(21) $$\begin{array}{l} \frac{\partial y_{o}}{\partial \underline{\alpha }}={\left[\frac{\partial y_{o}}{\partial \alpha_{1}},\;\frac{\partial y_{o}}{\partial \alpha_{2}},\;\cdots \frac{\partial y_{o}}{\partial \alpha_{\;i}},\;\ldots \;,\;\frac{\partial y_{o}}{\partial \alpha_{n{\;}_{i}}}\right]}^{T} \end{array}$$

(22) $$\begin{array}{l} \frac{\partial y_{o}}{\partial \underline{\beta }}={\left[\frac{\partial y_{o}}{\partial \beta_{1}},\;\frac{\partial y_{o}}{\partial \beta_{\;2}},\;\cdots ,\;\frac{\partial y_{o}}{\partial \beta_{\;i}},\;\ldots ,\;\frac{\partial y_{o}}{\partial \beta_{{\;}_{n{\;}_{i}}}}\right]}^{T} \end{array}$$

Then, by using the chain rule, the updating regulations of these two parameters can be expressed

(23) $$\begin{array}{l} \Delta \alpha_{i}=-\eta_{\alpha }\sum_{o=1}^{n_{o}}\frac{\partial E(k)}{\partial \alpha_{i}}\\ \;=-\eta_{\alpha }\sum_{o=1}^{n_{o}}\frac{\partial }{\partial y_{o}}\frac{1}{2}{\left(T_{o}-y_{o}\right)}^{2}\frac{\partial }{\partial \alpha_{i}}(a_{o}-p_{o})\;\\ \;=\eta_{\alpha }\sum_{o=1}^{n_{0}}e_{o}\left(w_{i\;o}-v_{i\;o}\right)\beta_{i}{\;}^{-1}(F_{i}{\;}^{2}-1)\exp\left(\frac{-F_{i}^{2}}{2}\right) \end{array}$$

(24) $$\begin{array}{l} \Delta \beta_{i}=-\eta_{\beta }\sum_{o=1}^{n_{o}}\frac{\partial E(k)}{\partial \beta_{i}}\\ \;=-\eta_{\beta }\sum_{o=1}^{n_{o}}\frac{\partial }{\partial y_{o}}\frac{1}{2}{\left(T_{o}-y_{o}\right)}^{2}\frac{\partial }{\partial \beta_{i}}(a_{o}-p_{o})\\ \;=\eta_{\beta }\sum_{o=1}^{n_{0}}e_{o}\left(w_{i\;o}-v_{i\;o}\right)\beta_{i}{\;}^{-1}F(F_{i}{\;}^{2}-1)\exp\left(\frac{-F_{i}^{2}}{2}\right) \end{array}$$

### Convergence Analysis

The following form is used to describe a class of uncertain nonlinear systems:

(25) $$\begin{array}{l} \left\{ \begin{array}{l} x^{\left(n\right)}(t)=f(\underline{x}(t))+G(\underline{x}(t))u(t)+d(\underline{x}(t))\\ \;=(f_{0}(\underline{x}(t))+\Delta f(\underline{x}(t))+(G_{0}(\underline{x}(t))+\Delta G(\underline{x}(t))u(t)\;\\ \;+\;d(\underline{x}(t))\;\\ \;=f_{0}(\underline{x}(t))+G_{0}(\underline{x}(t))+L(\underline{x}(t))\\ \;{\text{y}}_{s}\left(t\right)=x(t) \end{array}\right. \end{array}$$

where **x**(*t*), **u**(*t*), **d**(*t*) and **y**_s_ are the system state, the control signal, the external disturbance and the system output, respectively. The **x**(*t*) and **u**(*t*) can be defined as ${\;\left[\;x_{1}\left(t\right),x_{2}\left(t\right),\cdots ,x_{n_{o}}\left(t\right)\;\right]\;}^{T}$ and ${\;\left[\;u_{1}(t),u_{2}(t),\cdots ,u_{n_{o}}(t)\right]\;}^{T}$, belonging to ${ℜ}^{n_{o}}$. The term **x**(*t*) is thought to be obtainable, defined as ${\left[{\text{x}}^{T}(t),\;{\dot{\text{x}}}^{T}(t),\;\cdots \;,\;{\text{x}}^{(n\text{-}1)\;T}(t)\right]}^{T}$. Moreover, **f**(**x**(*t*))$\in {ℜ}^{n_{o}}$ is the system nonlinear vector, expressed as **f**_0_(**x**(*t*)) + Δ**f**(**x**(*t*)) and **G**(**x**(*t*))$\in {ℜ}^{n_{o}\;\times \;n_{o}}$ is the matrix-valued function, expressed as **G**_0_(**x**(*t*)) + Δ**G**(**x**(*t*)). Here, the second parts of them are the uncertain functions of the system whose boundaries are assumed to be obtainable, and the inverse of *G*_0_(**x**(*t*)) exists. Consequently, the unknown uncertainty of the system is described by these unknown items in Equation (25), i.e.**Δf(x(t))**, **Δ***G*(**x(t))u(t)**, and **d**(**t**), represented as **L(x(t))** ∈ ℜ^**n**_**o**_^. Its boundary is also thought to be obtainable.

Here, we define a tracking error as *e*(*t*) = *y*_*d*_(*t*) − *y*_*s*_(*t*), its vector form is

(26) $$\begin{array}{l} \underline{e}(t)\;={\left[e\;(t){\;}^{T},\;e^{\prime }\;(t){\;}^{T},\;...\;,\;e\;(t){\;}^{\left(n-1\right)}{\;}^{T}\right]}^{\;T}\in {ℜ}^{n{\;}_{i}\;n{\;}_{o}} \end{array}$$

A sliding hyper-plane is used to express the integrated error function

(27) $$\begin{array}{l} s(\underline{e},t)\equiv \;e^{\left(n-1\right)}+K_{1}e^{\left(n-2\right)}+...+K_{n}\int_{\;0}^{\;t}e(\tau )d\tau \end{array}$$

where ${\text{K}}_{i}\in {ℜ}^{n_{o}\times n_{o}}$, *i* = 1, 2, …, *n*_*i*_, are matrices with positive constant and define **K** = [K_1_^*T*^, …, K_n_^*T*^]^*T*^ $\in {ℜ}^{n_{i}n_{o}\times n_{o}}$,$\text{s}\left(\underline{\text{e}},t\right)={\left[s_{1}\left(t\right),s_{2}\left(t\right),\cdots \;,s_{n_{o}}\left(t\right)\right]}^{T}\in {ℜ}^{n_{o}}$ is a sliding surface vector.

When the uncertainty and the nominal functions, such as *L*(**x**(*t*)), **f**_0_(**x**(*t*)) and **G**_0_(**x**(*t*)), are really known, we can design an ideal controller as

(28) $$\begin{array}{l} u^{*}(t)=G_{0}{\;}^{-1}(\underline{x}(t))\;\left[y_{d}{\left(t\right)}^{\left(n\right)}-f_{0}(\underline{x}(t))-L(t)+K^{T}\underline{e}\right] \end{array}$$

A dynamic equation of the tracking error can be given after replacing (Equation 25) with the ideal controller shown in Equation (28)

(29) $$\begin{array}{l} \dot{s}(\underline{e},t)=e^{\left(n\right)}+K^{T}\underline{e} \end{array}$$

By selecting the value of **K** in Equation (29), the roots of the polynomial can locate in the left half of the complex plane. It means that with the time goes by, the tracking error will become smaller and smaller until it converges to zero. In practice, however, this uncertainty **L**(**x**(*t*)) is usually not obtained, so the ideal controller u^*^(*t*) in Equation (28) can not be implemented.

Therefore, a WFBELC controller and a robust controller are needed to be combined into a WFBELC control system to deal with this problem.

(30) $$\begin{array}{l} u\;=u_{\;WFBELC}+u{\;}_{r} \end{array}$$

Thus, in this proposed WFBELC control system, there are a main controller **u**_WFBELC_ and a robust compensator controller **u**_r_. The **u**_WFBELC_ is utilized to approach the ideal controller u^*^(*t*), while the function of the compensator controller is to compensate the approximation error ε between u^*^(*t*) and **u**_WFBELC_.

Then, a **L**_WFBELC_ would be appeared to approximate the **L**(x(t)) according to the universal approximation theorem (Wang, 1994).

(31) $$\begin{array}{l} L(\underline{x}(t))=L_{WFBELC}(I,w_{i\;o},v_{i\;o},m_{i},\sigma_{i})+\varepsilon \end{array}$$

Take the derivative of Equation (27) and use (Equation 25), then

(32) $$\begin{array}{l} \dot{s}(\underline{e},t)=e^{\left(n\right)}+K^{T}\underline{e}\\ \;=-f_{0}(\underline{x}(t))-G_{0}(\underline{x}(t))\;u(t)+y_{d}{\left(t\right)}^{\left(n\right)}\\ \;-L_{WFBELC}(\underline{x}(t))+K^{T}\underline{e} \end{array}$$

Substituting (Equation 30) into (Equation 32), multiplying each side by *s*^*T*^ gives

(33) $$\begin{array}{l} s^{T}\dot{s}=-s^{T}f_{0}(\underline{x}(t))-s^{T}G_{0}(\underline{x}(t))\;(u_{\;WFBELC}(t)+u{\;}_{r}(t))\\ \;+s^{T}(y_{d}{\left(t\right)}^{\left(n\right)}-L_{WFBELC}(\underline{x}(t))+K^{T}\underline{e}) \end{array}$$

where

(34) $$\begin{array}{l} u_{WFBELC}(t)=G_{0}{\;}^{-1}(\underline{x}(t))[\;y_{d}{\left(t\right)}^{\left(n\right)}-f_{0}(\underline{x}(t))-L_{WFBELC}(\underline{x}(t))\\ \;\;+K^{T}\underline{e}] \end{array}$$

In the course of observation, the approximation error ε in Equation (31) is assumed to be bounded and the boundary is difficult to obtain. Therefore, we define an estimated value to estimate the boundary of this error.

(35) $$\begin{array}{l} \tilde{N}=N-\hat{N} \end{array}$$

where *N* is the boundary, whose estimated value is $\hat{N}$. Then, it can be assumed to bound ε ∈ [0, *N*].

Hence, the compensator in Equation (30) can be selected as

(36) $$\begin{array}{l} u_{r}=-G_{0}^{-1}(\underline{x})\hat{N}\text{sgn}(s) \end{array}$$

And (Equation 32) is also rewritten as

(37) $$\begin{array}{l} \dot{s}(\underline{e},t)=e^{\left(n\right)}+K^{T}\underline{e}=\;G_{0}(\underline{x})u_{r}(t)+\varepsilon \end{array}$$

In this paper, a Lyapunov function is given as

(38) $$\begin{array}{l} \Gamma (s,\tilde{N})=\frac{s^{T}s}{2}+\frac{{\tilde{N}}^{2}}{2\eta_{N}} \end{array}$$

where η_*N*_ is the learning rate of Ñ, whose adaptive learning law is selected as

(39) $$\begin{array}{l} \dot{\hat{N}}=-\eta_{N}\left|\;s\;\right| \end{array}$$

Because of the constant value, $\dot{Ñ}=-\dot{\hat{N}}$.

Taking the derivative of Equation (38), and using (Equations 36, 37), then

(40) $$\begin{array}{l} \dot{\Gamma }(s,\tilde{N})=s^{T}\dot{s}+\frac{\tilde{N}\dot{\tilde{N}}}{\eta_{N}}=s^{T}(\varepsilon -\hat{N}\text{sgn}(s))+\frac{\tilde{N}\dot{\tilde{N}}}{\eta_{N}}=s^{T}\varepsilon -\hat{N}\left|s\right|\\ \;+\frac{\tilde{N}\dot{\tilde{N}}}{\eta_{N}} \end{array}$$

Substituting (Equations 35, 40) into (Equation 38), $\dot{\Gamma }(s\text{,}\tilde{N}(t)$ becomes

(41) $$\begin{array}{l} \dot{\Gamma }(s,\tilde{D})=s^{T}\varepsilon -\hat{N}\left|s\right|-(N-\hat{N})\left|s\right|=s^{T}\varepsilon -N\left|s\right|\\ \;\leq \left|\varepsilon \right|\;\left|s\right|-N\left|s\right|\;=-(N-\left|\varepsilon \right|)\left|s\right|\leq 0 \end{array}$$

Since $\dot{\Gamma }(s\text{,}\tilde{N}(t)$ is negative semidefinite function, i.e., $\dot{\Gamma }(s(t),Ñ(t)\text{)}\leq \dot{\Gamma }(s(0),Ñ(0)\text{)}$, it indicates **s** and Ñ are bounded.

(42) $$\begin{array}{l} \left(N-\left|\varepsilon \right|)\;s\leq (N-\left|\varepsilon \right|)\left|s\right|\leq -\dot{\Gamma }(s,\tilde{N}\right) \end{array}$$

Let function Ξ = (*N* − |ε|)**s**, and integrate Ξ(*t*), obtains

(43) $$\begin{array}{l} \int_{\;0}^{\;t}\Xi (\tau )d\tau \leq \Gamma (s(0),\tilde{N}(0))-\Gamma (s(t),\tilde{N}(t)) \end{array}$$

Because Γ(**s**(0), Ñ(0)) is bounded, and Γ(**s**(*t*), Ñ(*t*)) is non-increasing and bounded, we derive:

(44) $$\begin{array}{l} \underset{t\;\to \;\infty }{\lim}\int_{\;0}^{\;t}\Xi (\tau )d\tau \leq \infty \;,(t\;\to \;\infty ,\;s(t)\;\to 0\;) \end{array}$$

In addition, according to Barbalat's lemma (Slotine and Li, 1991), since $\dot{\Xi }\left(t\right)$ possesses the boundary, Ξ(*t*) → 0 when *t* → ∞. Hence, the WFBELC system possesses the asymptotic stability and satisfactory convergence. The tracking error will approximate to zero in virtue of *t* → ∞. A favorable robust tracking performance can be achieved consequently.

Moreover, the actual system and mathematical model are not exactly matched. To solve this problem, the uncertainty of these mathematical functions can be used to include these mis-matches. As a result, this WFBELC control system can be used for nonlinear systems with uncertainties to enhance their robust control characteristics.

## Simulation Results

Two chaotic systems are used as the cases to verify the effectiveness of the presented WFBELC mode applied for uncertain nonlinear systems. For comparison, some other models are also applied.

### Duffing-Holmes Chaotic System

Chaotic system is a kind of uncertain nonlinear systems, which is complex and unpredictable. Its disorder randomness is derived from the nonlinear term in the internal dynamics equation. One of the characteristics of chaotic systems is their high sensitivity to initial conditions, such that even very tiny differences in initial conditions may have a great impact on the behavior of these systems (Chang and Yan, 2005). The Duffing-Holmes chaotic system is considered. It is a second-order chaotic system expressed as Yan et al. (2005):

(45) $$\begin{array}{l} \{\begin{array}{l} {\dot{x}}_{1}(t)=x_{2}(\text{t})\\ {\dot{x}}_{2}(t)=-p_{1}\;x_{1}(t)-p_{2}x_{1}(t){\;}^{3}-p_{3}x_{2}(t)+q\cos(\omega \;t)\\ y_{s}(y)=x_{1}(t) \end{array} \end{array}$$

where *p*_1_ is the damping coefficient, *q*cos(ω*t*) is the periodic driving signal and *q* is the amplitude.

**Figure 2:**
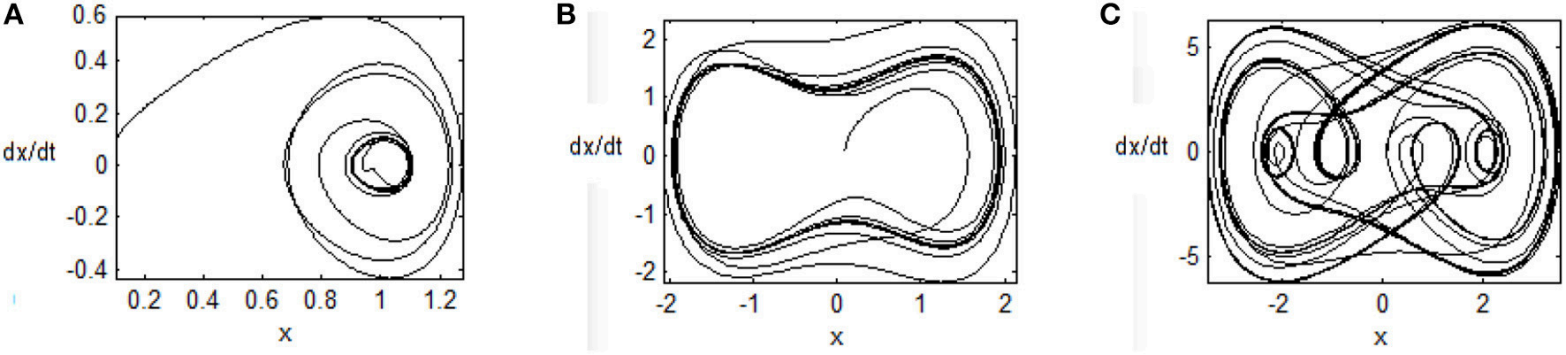
Phase plane of uncontrolled chaotic system **(A)** *q* = 0.1, **(B)** *q* = 1, and **(C)** *q* = 8.

For different *q* values, the trajectories of the chaotic system are different, just as seen in Figures 2A–C with the real constants [*p*_1_, *p*_2_, *p*_3_] = [−1, 1, 0.25]. When *q* = 0.1, the trajectory is centered around a point (1, 0). When *q* = 1, the chaotic phenomenon appears, and the trajectory is periodically run around two points, (−1, 0) and (1, 0). When *q* = 8, besides the above characteristics, the trajectory is sunken toward the interior of orbit and cross state is emerged.

When we take into account the unknown factors, the external interference signals, and the system control signals, a dynamic Equation (46) is given:

(46) $$\begin{array}{l} \{\begin{array}{l} {\dot{x}}_{1}(t)=x_{2}(\text{t})\\ {\dot{x}}_{2}(t)=-p_{1}\;x_{1}(t)-p_{2}x_{1}(t){\;}^{3}-p_{3}x_{2}(t)+q\cos(\omega \;t)\\ \;+\Delta f(X,t)+d(t)+u(t)\\ y_{s}(t)=x_{1}(t) \end{array} \end{array}$$

where the uncertainty term Δ*f*(*X, t*) and the disturbance *d*(*t*) are selected as $0.1\;\times sqrt\left({x_{1}}^{2}+{x_{2}}^{2}\right)\times \sin\left(t\right)$ and 0.1 × sin(*t*).

**Figure 3:**
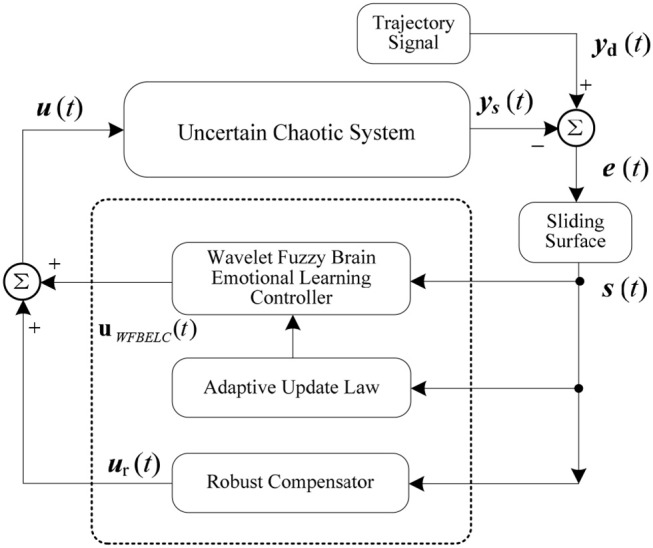
Adaptive WFBELC control for uncertain chaotic system.

Then, an adaptive WFBELC control for uncertain chaotic system is built in Figure 3.

For the example, the initial values of a sliding hyper-plane are selected as $K_{1}=\left[\begin{array}{cc} k_{11} & 0\\ 0 & k_{12} \end{array}\right]$, *k*_11_ = 0.6, *k*_12_ = 0.02, which are based on the stable integrated error function in Equation (27). The dilation parameter for a wavelet function is set as β_i_ = 3, and the translation parameter for a wavelet function is distributed in the interval [-2, 2].

Choosing an appropriate learning rate for the parameter updating law is one of the most important aspects of network design. Here, the learning rates in the amygdala and in the prefrontal cortex, η_*v*_ and η_*w*_, are both equal to 0.01 according to Zhao and Lin (2017). The constant gains in Equation (15) are selected as λ_i_ = 60, (*i* = 1, 2), γ_*o*_ = 1.2, (*o* = 1). The compensator parameter η_*N*_ = 0.1, $\hat{N}=1$. These parameters are selected based on some trial-error method to ensure the required transient performance of this control system. Other parameters are random.

**Figure 4:**
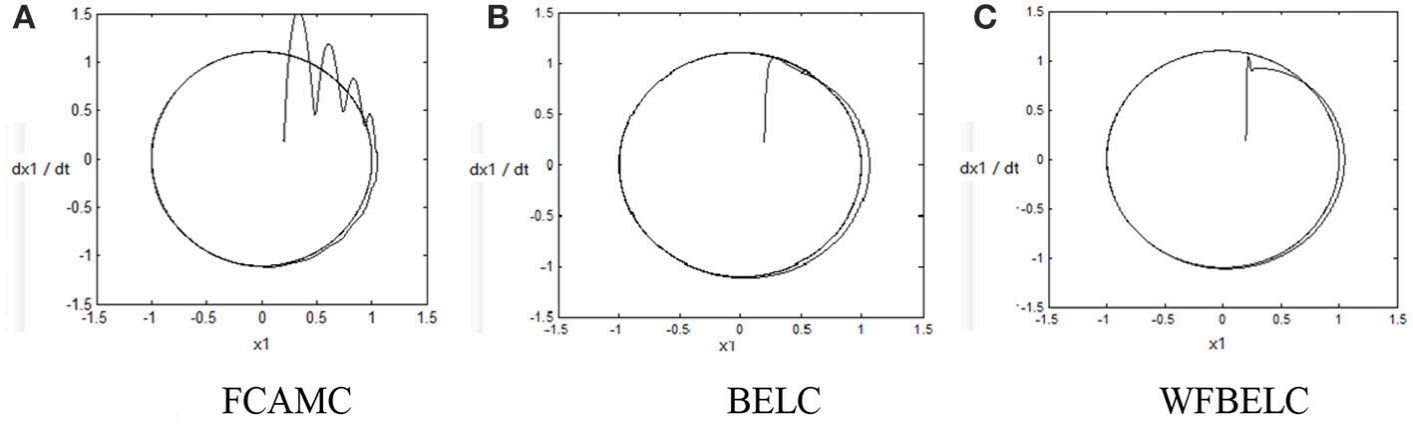
Periodic orbit phase plane plots (*q* = 0.3) **(A)** FCAMC, **(B)** BELC, and **(C)** WFBELC.

**Figure 5:**
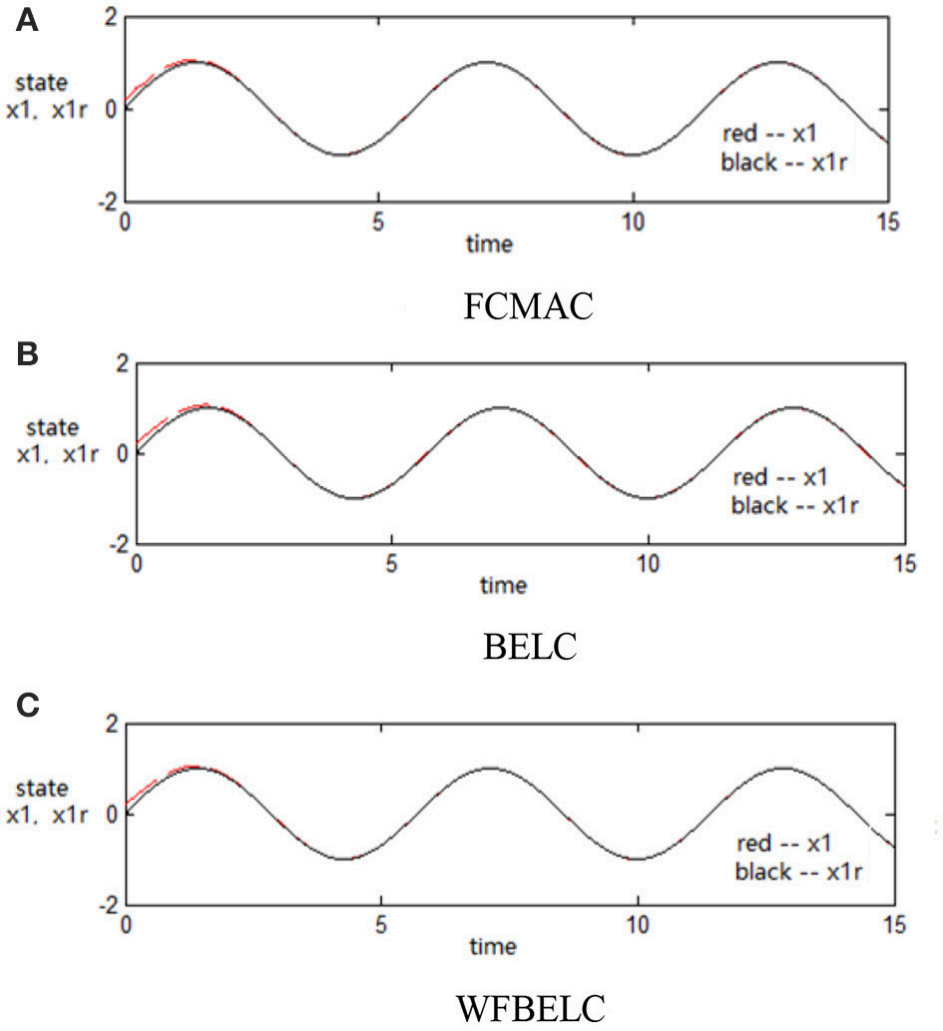
Response tracking curves of state *x*_1_ (*q* = 0.3) **(A)** FCMAC, **(B)** BELC, and **(C)** WFBELC.

**Figure 6:**
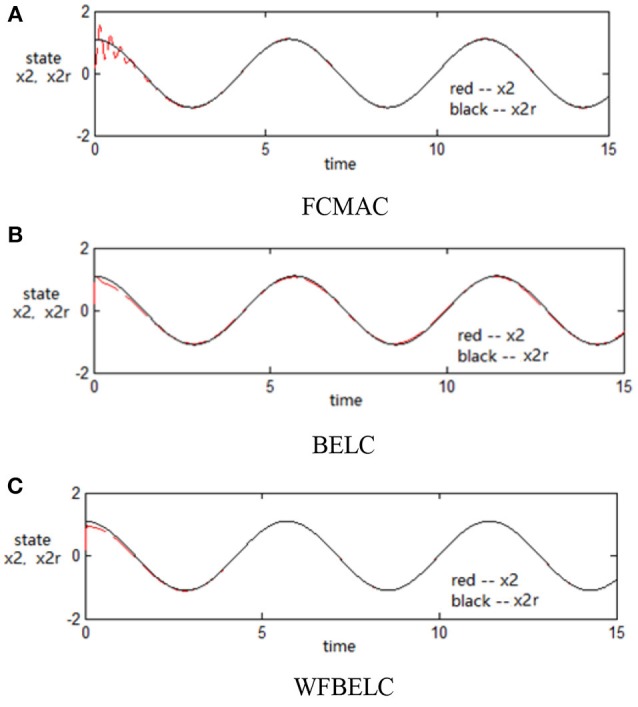
Response tracking curves of state *x*_2_ (*q* = 0.3) **(A)** FCMAC, **(B)** BELC, and **(C)** WFBELC.

**Figure 7:**
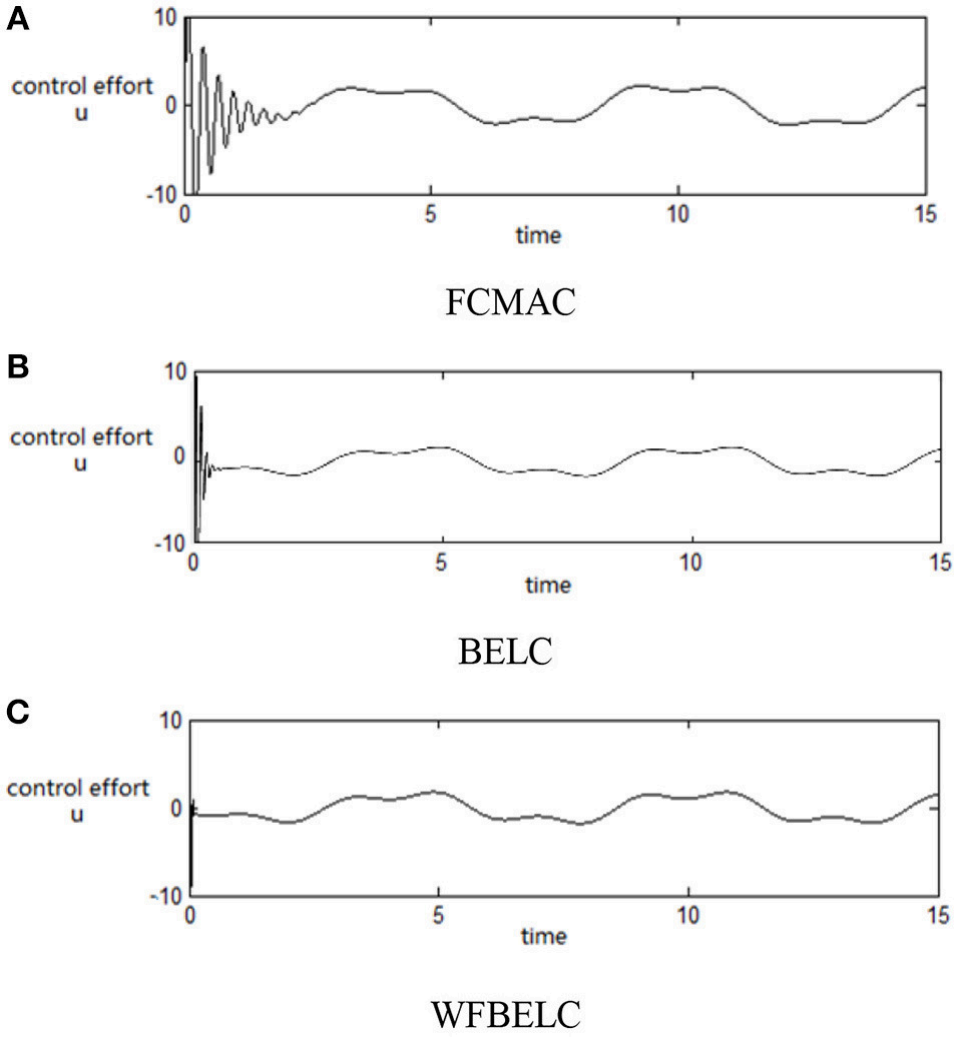
Change trend chart of the control signal *u* (*q* = 0.3) **(A)** FCMAC, **(B)** BELC, and **(C)** WFBELC.

**Figure 8:**
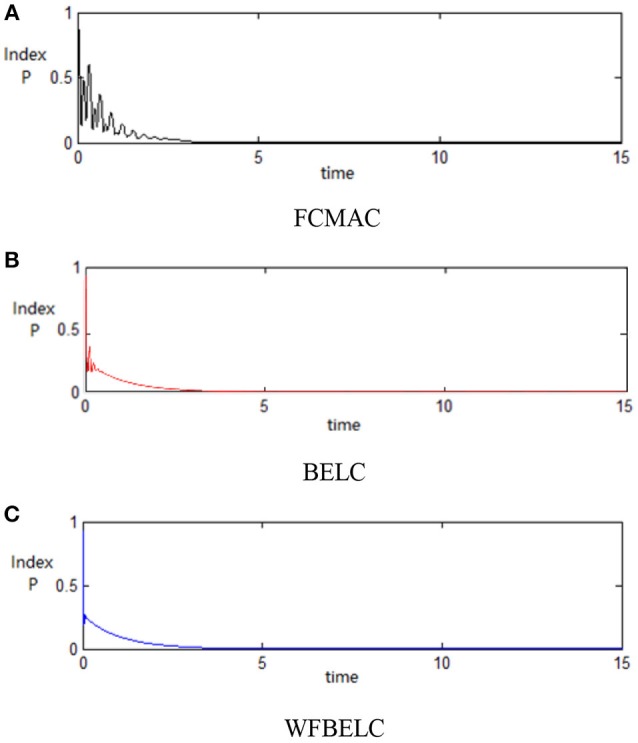
Performance Index *P* (*q* = 0.3) **(A)** FCMAC, **(B)** BELC, and **(C)** WFBELC.

**Figure 9:**
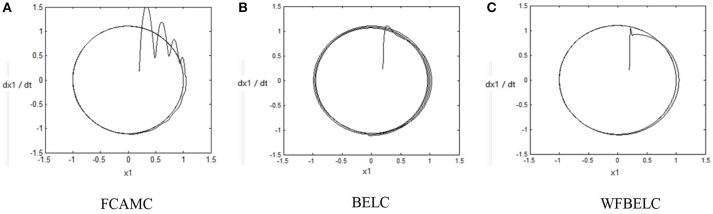
Periodic orbit phase plane plots (*q* = 8) **(A)** FCAMC **(B)** BELC **(C)** WFBELC.

**Figure 10:**
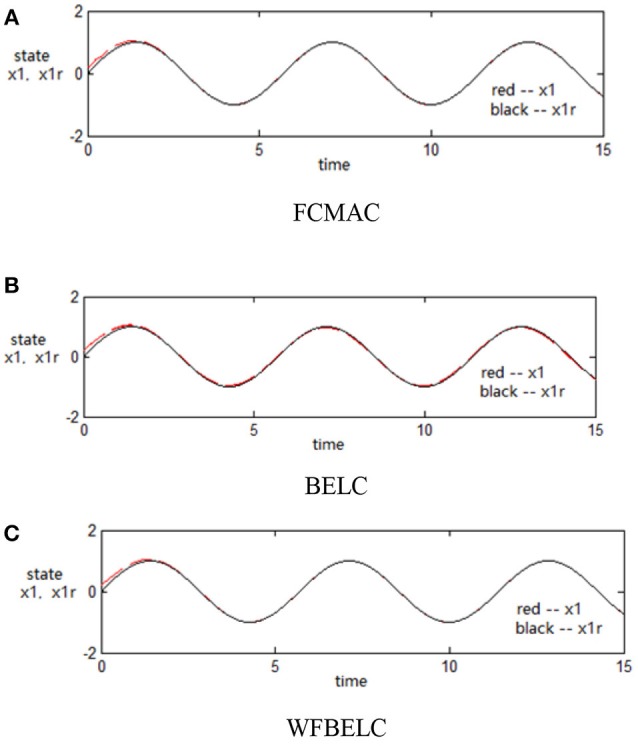
Response tracking curves of state *x*_1_ (*q* = 8) **(A)** FCMAC, **(B)** BELC, and **(C)** WFBELC.

**Figure 11:**
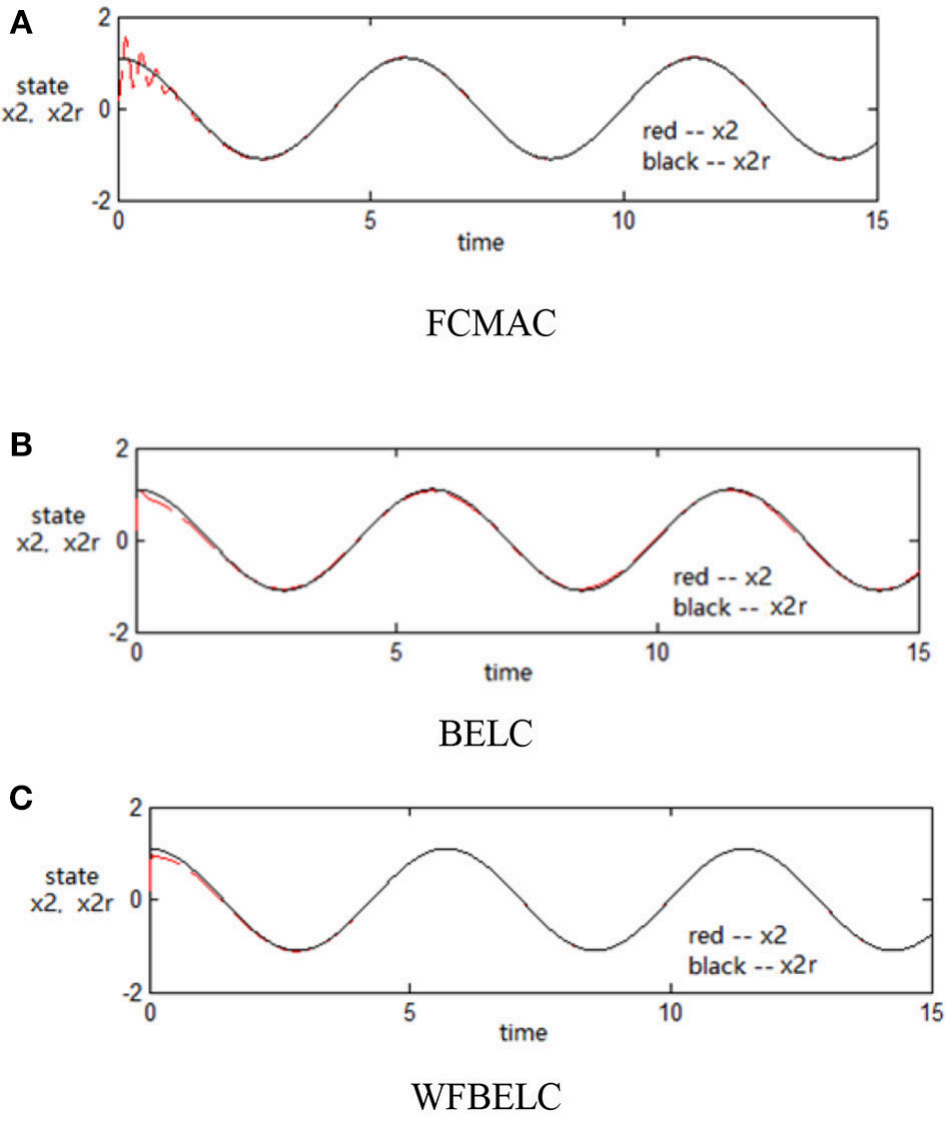
Response tracking curves of state *x*_2_ (*q* = 8) **(A)** FCMAC, **(B)** BELC, and **(C)** WFBELC.

**Figure 12:**
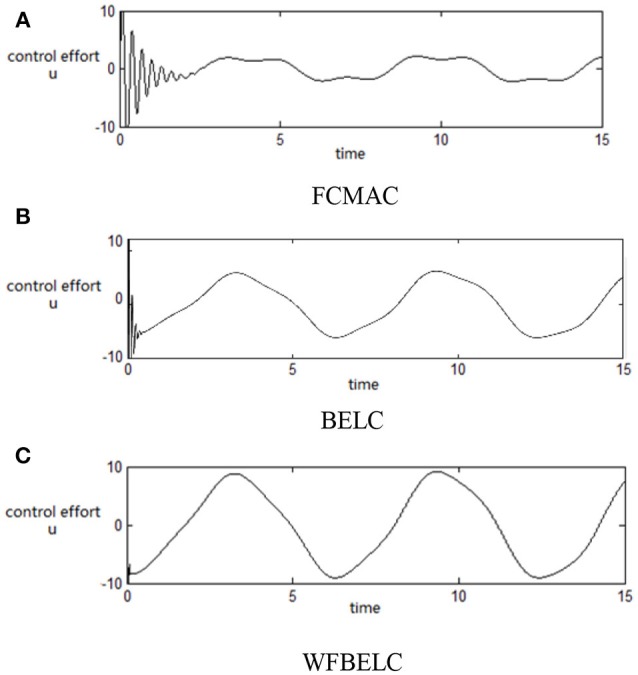
Change trend chart of the control signal *u* (*q* = 8) **(A)** FCMAC, **(B)** BELC, and **(C)** WFBELC.

**Figure 13:**
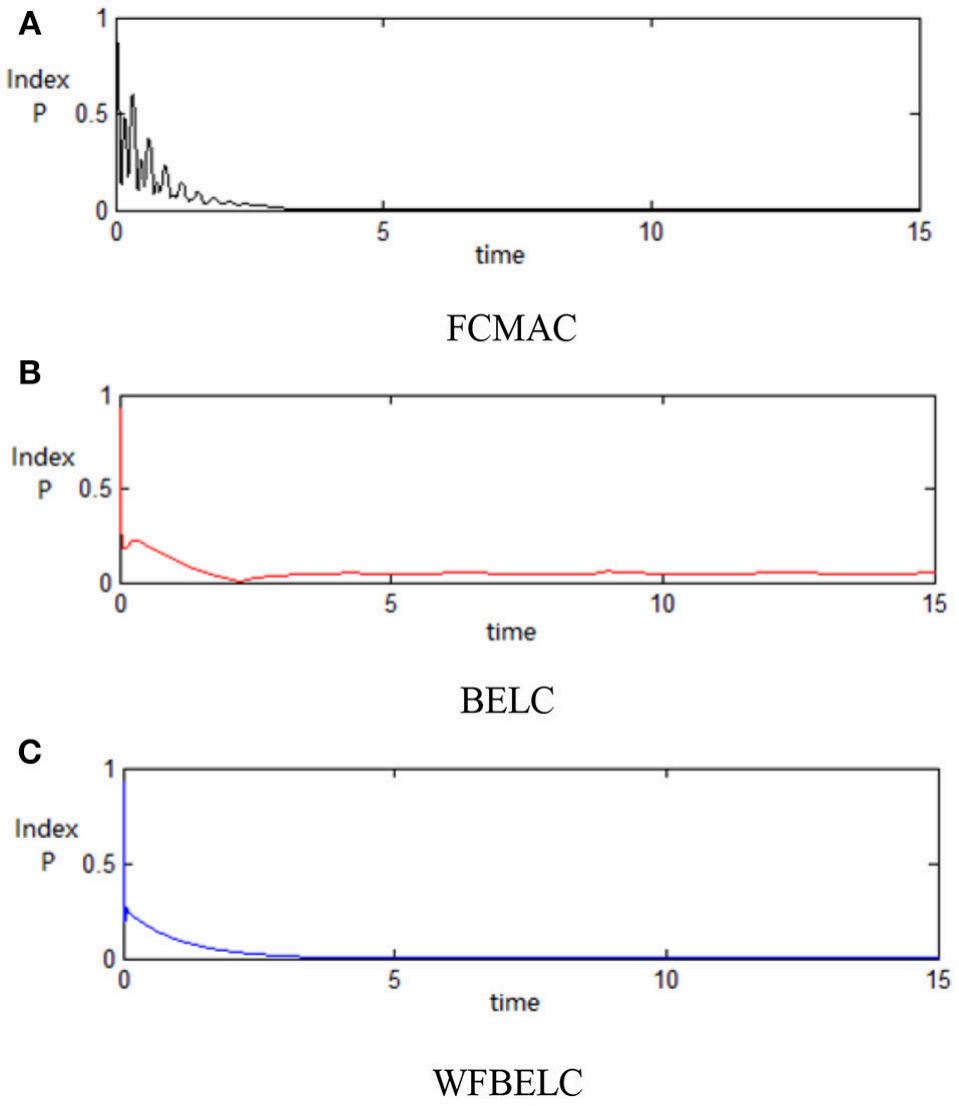
Performance Index *P* (*q* = 8) **(A)** FCMAC, **(B)** BELC, and **(C)** WFBELC.

For the sake of verifying the effectiveness of WFBELC, an FCMAC (El-Sousy and Abuhasel, 2016) and a BELC (Chung and Lin, 2014) are also applied with different *q* value in Equation (46). A performance index *P* is defined as $P=\sqrt{e^{2}+{e^{\prime }}^{2}}$. The trajectory signal is set as *y*_*d*_(*t*) = *x*_1_(*t*) = sin(1.1 *t*). Simulation results with *q* = 0.3 of FCMAC, BELC and WFBELC are respectively depicted in Figures 4–8, namely: the periodic orbit phase plane plots, the response tracking curves of state *x*_1_ and state *x*_2_, the change trend chart of the control signal *u* and performance Index *P*. For the case of *q* = 8, there are also the set of such diagrams depicted in Figures 9–13. The values of root mean square error (RMSE) and the computation cost of this chaotic system using different models are listed in Table 1.

**Table 1:** Results for Duffing-Holmes chaotic system.

		**FCMAC**	**BELC**	**WFBELC**
RMSE	*q* = 0.3	0.062193	0.05008	0.047998
	*q* = 8	0.077768	0.067314	0.050479
Computation time (s)	*q* = 0.3	1.3715	1.2643	1.1806
	*q* = 8	1.4021	1.3286	1.2968

According to the above compared results, for different chaotic trajectories, much more satisfactory control performance can be obtained by using the presented WFBELC than by using the FCMAC or by using the BELC. The more serious the chaos is, the better the tracking performances of the WFBELC are. The control system with WFBELC model structure has smaller tracking error than the control system with other two model structures. Moreover, the convergence speed of the WFBELC model is also the fastest compared with the other two models. From Table 1, it can be seen that there are more parameters to upgrade the effectiveness of WFBELC control system than those of the other two control systems, so it costs a little more time in computation. This increased computation time is still acceptable.

### Chua's Chaotic Circuit

**Figure 14:**
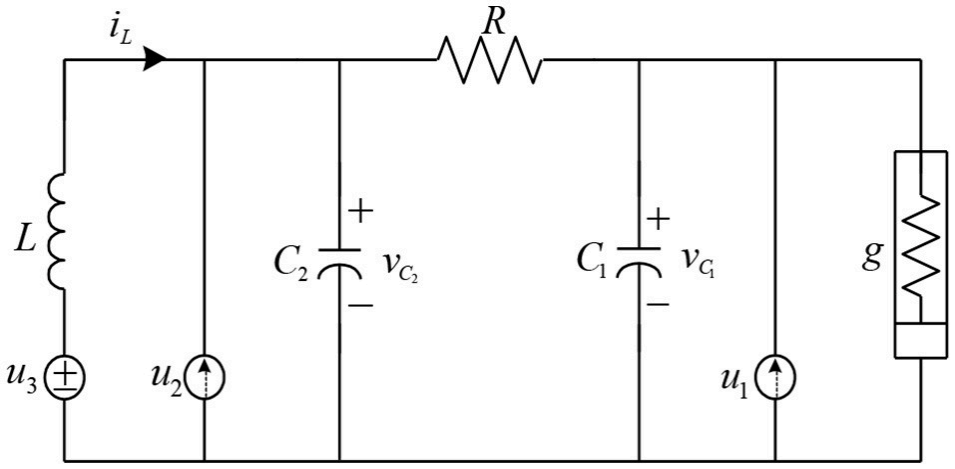
Chua's chaotic circuit.

A typical kind of the Chua's chaotic circuit structure is shown in Figure 14.

Then the dynamic equations for this circuit are given as Chang and Robust (2004).

(47) $$\begin{array}{l} {\dot{v}}_{C_{1}}=\frac{1}{C_{1}}\left(\frac{1}{R}\left(v_{C_{2}}-v_{C_{1}}\right)-g\left(v_{C_{1}}\right)+u_{1}\left(t\right)\right)+d_{1}\left(t\right) \end{array}$$

(48) $$\begin{array}{l} {\dot{v}}_{C_{2}}=\frac{1}{C_{2}}\left(\frac{1}{R}\left(v_{C_{1}}-v_{C_{2}}\right)+i_{L}+u_{2}\left(t\right)\right)+d_{2}\left(t\right) \end{array}$$

(49) $$\begin{array}{l} {\dot{i}}_{L}=\frac{1}{L}\left(-v_{C_{2}}+u_{3}\left(t\right)\right)+d_{3}\left(t\right) \end{array}$$

where *R, g, C*_1_, *C*_2_, and *L* are the physical parameters, i.e., the linear resistor, the nonlinear resistor, the capacitor and the inductor. The$\;\text{d}\left(t\right)={\left[d_{1}\left(t\right),d_{2}\left(t\right),d_{3}\left(t\right)\right]}^{T}$ expresses the disturbance signal and $\;\text{u}\left(t\right)={\left[u_{1}\left(t\right),u_{2}\left(t\right),u_{3}\left(t\right)\right]}^{T}\;$expresses the control signal. The *v*_*C*1_(*t*), *v*_*C*2_(*t*) and the *i*_*L*_(*t*) are the state variables of the voltages and the current for this chaotic circuit. Thus, the input state vector of this circuit can be given as $\text{x}\left(t\right)={\left[x_{1}\left(t\right),x_{2}\left(t\right),x_{3}\left(t\right)\right]}^{T}={\left[v_{C_{1}}\left(t\right),v_{C_{2}}\left(t\right),i_{L}\left(t\right)\right]}^{T}$. These aforementioned dynamic equations are re-given as

(50) $$\begin{array}{l} x^{\left(n\right)}(t)=f(\underline{x}(t))+G(\underline{x}(t))u(t)+d(\underline{x}(t))\\ Here,f\left(\underline{x}\right)=\left[\begin{array}{l} \frac{1}{C_{1}}\left(\frac{1}{R}\left(v_{C_{2}}-v_{C_{1}}\right)-g\left(v_{C_{1}}\right)\right)\\ \;\frac{1}{C_{2}}\;\left(\frac{1}{R}\left(v_{C_{1}}-v_{C_{2}}\right)+i_{L}\right)\\ \;\frac{1}{L}\left(-v_{C_{2}}\right) \end{array}\right] \end{array}$$

and

$$G\left(\underline{x}\right)=diag\left[\begin{array}{lll} \frac{1}{C_{1}} & 0 & 0\\ 0 & \frac{1}{C_{2}} & 0\\ 0 & 0 & \frac{1}{L} \end{array}\right]$$

The external disturbance is given as

(51) $$\begin{array}{l} d\left(t\right)=\left[\begin{array}{c} d_{\text{1}}\left(t\right)\\ d_{\text{2}}\left(t\right)\\ d_{\text{3}}\left(t\right) \end{array}\right]=\left[\begin{array}{c} exp\left(-t/5\right)\;sin\;\left(2t\right)+0.3\;\\ exp\left(-t/5\right)\;cos\;\left(2t\right)\;-0.5\\ exp\left(-t/5\right)\;sin\;\left(3t\right)+0.2 \end{array}\right] \end{array}$$

The parameters of the resistance, inductance, and capacitance are formed as *R* = *R*_0_+Δ*R, g*(*v*_*C*__1_) = *g*_0_(*v*_*C*__1_)+Δ*g*(*v*_*C*__1_),*L* = *L*_0_ +Δ*L*,*C*_1_ = *C*_10_+ΔC_1_,*C*_2_ = *C*_20_+ΔC_2_, where *R*_0_, *g*_0_(*v*_*C*__1_), *L*_0_, *C*_10_, and *C*_20_ are the nominal values and Δ*R*, Δ*g*(*v*_*C*__1_), Δ*L*, ΔC_1_, and ΔC_2_ represent the unknown nonlinear time-varying perturbations. The nominal values are set as *R*_0_ = 5, $g_{0}\left(v_{C_{1}}\right)=-v_{C_{1}}+0.02v_{C_{1}}^{3}$, *L*_0_ = 1, *C*_10_ = 1, and *C*_20_ = 0.5. The time-varying perturbations are Δ*R* = sin(0.5*t*), Δ*g*(*v*_*C*__1_) = 0.2 *v*_*C*__1_×sin(*t*), Δ*L* = 0.15, ΔC_1_ = 0.1cos(0.5 *t*) +0.1 and ΔC_2_ = 0.1. The required trajectories are *x*_*r*_ = [*x*_*r*1_, *x*_*r*2_, *x*_*r*3_] ^*T*^ = [1/5 × sin(3*t*)+sin(*t*), 1/5 × cos(3*t*)+cos(*t*), sin(*t*)+1] ^*T*^ (Joe et al., 2014). The initial values of state parameters, like the system states and the reference model states, are set as *x*_1_(0) = 0, *x*_2_(0) = −1, *x*_3_(0) = 0; *x*_*r*1_(*t*) = 0, *x*_*r*2_(*t*) = 1, *x*_*r*3_(*t*) = −1. A sliding hyper plane, **s**(e, *t*) = **e**(*t*), is proposed as for the proposed control case.

For the proposed WFBELC control system, the input signals of WFBELC are *e*_1_(*t*), *e*_2_(*t*), and *e*_3_(*t*), the initial parameters of sliding mode are set as **K**_1_ = diag(0.1, 0.1, 0.1) based on Equation (27). The dilation parameter for a wavelet function is set as β_i_ = 1.6 and the translation parameter for a wavelet function is distributed in the interval [−2, 2].The learning rates in the sensory cortex space are selected as η_*v*_ = η_*w*_ = 0.15. The other parameters are set as same as in Example 1.

**Figure 15:**
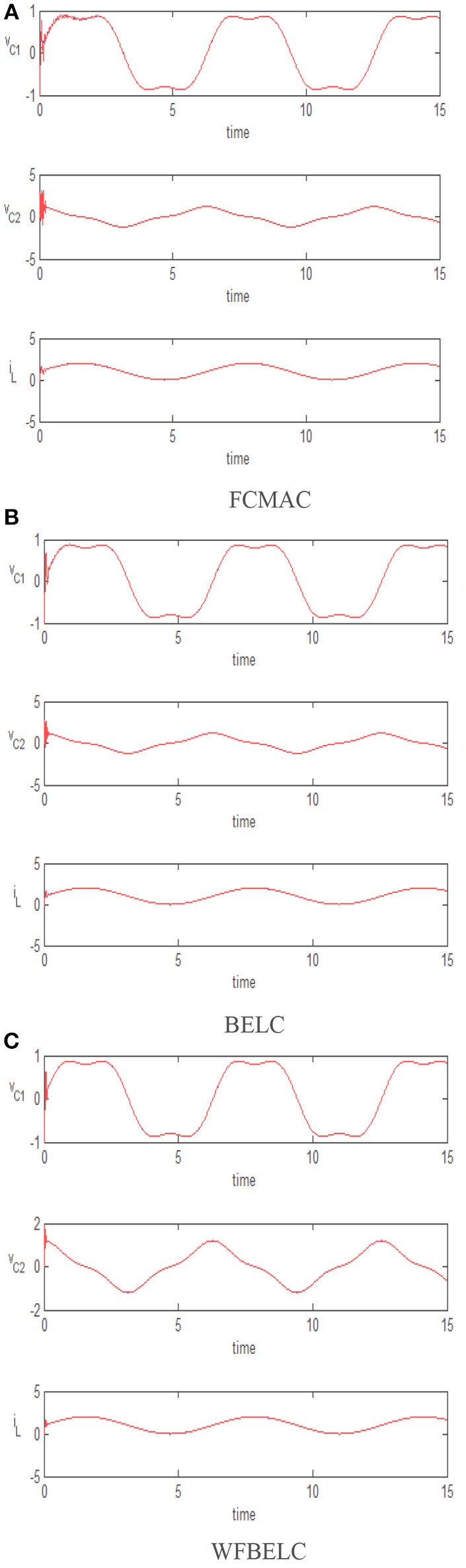
Trajectory responses **(A)** FCMAC, **(B)** BELC, and **(C)** WFBELC.

**Figure 16:**
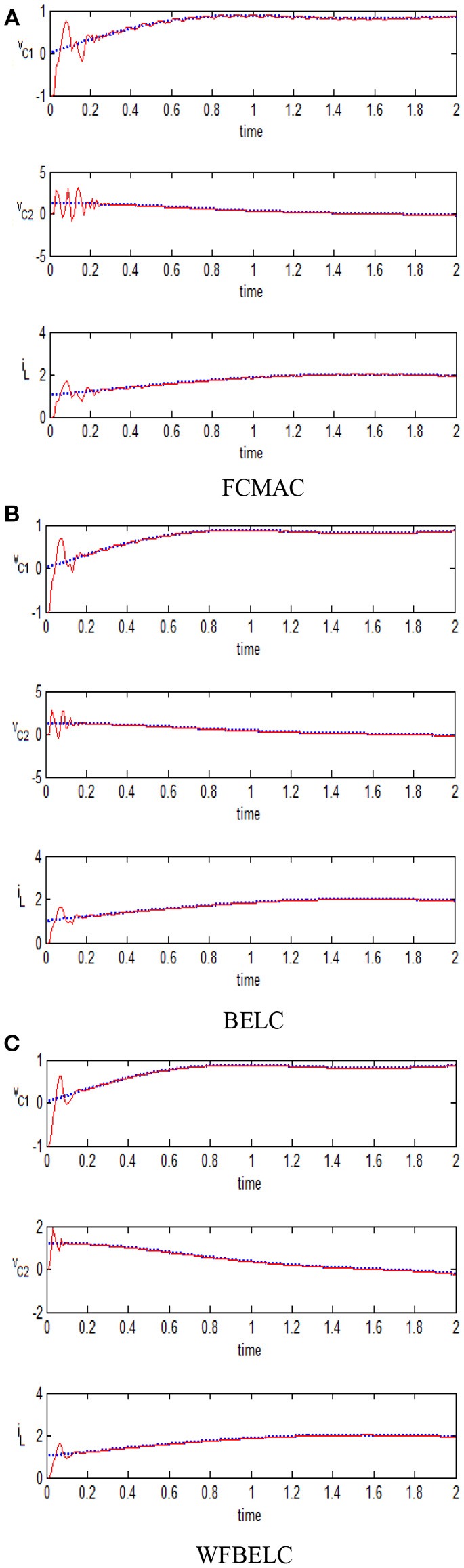
Enlarged responses **(A)** FCMAC, **(B)** BELC, and **(C)** WFBELC.

**Figure 17:**
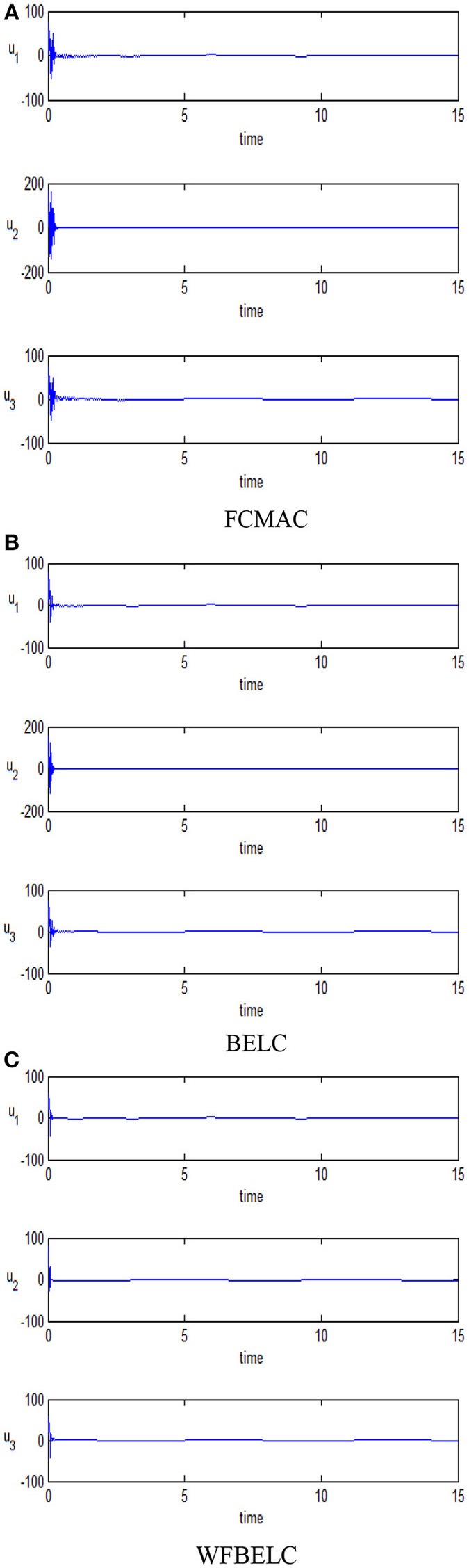
Corresponding control inputs **(A)** FCMAC, **(B)** BELC, and **(C)** WFBELC.

**Figure 18:**
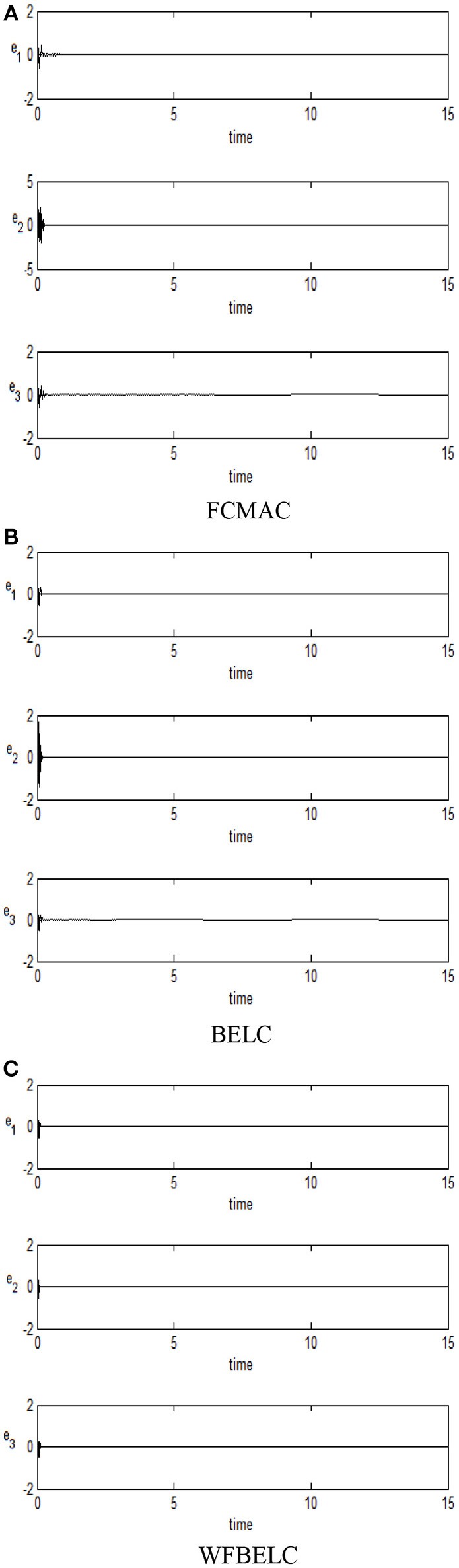
Values of tracking error **(A)** FCMAC, **(B)** BELC, and **(C)** WFBELC.

For comparison, a FCMAC and a BELC are also applied. The simulation results of this chaotic example are plotted as follows: the trajectory responses [*v*_*C*1_(*t*), *v*_*C*2_(*t*), *i*_*L*_(*t*)] are plotted in Figure 15; the enlarged responses are plotted in Figure 16, the dotted lines represent the reference signals and the red lines represent the responses; the corresponding control inputs [*u*_1_(*t*), *u*_2_(*t*), *u*_3_ (*t*)] are plotted in Figure 17; and the values of tracking error [*e*_1_(*t*), *e*_2_(*t*), *e*_3_ (*t*)] are plotted in Figure 18. The RMSE and the computation cost of these systems are tabulated in Table 2.

**Table 2:** Results for Chua's chaotic circuit.

		**FCMAC**	**BELC**	**WFBELC**
RMSE	*e*_1_	0.051962	0.047958	0.043589
	*e*_2_	0.143875	0.098489	0.042426
	*e*_3_	0.05099	0.046904	0.042426
Computation time (s)		1.0279	1.0071	1.1371

In these simulations, from the Figures 15–18 and Table 2, the WFBELC control system can achieve faster errors convergence and reduce tracking error to get smaller tracking errors than the FCMAC control system and the traditional BELC control system. The tracking error convergence speed of the WFBELC is also faster than that of these two other systems. Moreover, it obtains obviously from Table 2 that for the same chaotic circuit, the RMSE of the WFBELC control system is smaller than the other two control systems. Similar to the previous example, compared with other models, the computation time has increased slightly by using the WFBELC model to improve the effectiveness of WFBELC control system. It is acceptable, as well.

## Conclusions

The main contribution of this paper is to design the WFBELC model, which can be applied to much more effectively solve the uncertainty of the MIMO nonlinear systems. It consists of the wavelet theory, the type −1 fuzzy inference and the BEL algorithm. Thus, the WFBELC model has the advantages of them such that it can mimic the expression of the brain's sensations and emotions in one, and can describe the complex uncertain nonstationary signals more detailed. When the WFBELC is used as the main tracking controller for a MIMO uncertain nonlinear system and the robust compensation controller is used as a compensator, the control performances can be improved. Simulation results of two chaotic systems confirm that this proposed WFBELC model can effectively obtain satisfactory control capability with better transient responses and smaller error values comparing to the FCMAC control scheme and the BELC control scheme. Finally, the satisfactory control performance can be obtained much more quickly and effectively than other schemes. These comparisons also show that the proposed model can be more capable to handle the influence of the uncertainty. Moreover, the proposed control method can also be suitable for a large class of unknown nonlinear systems, because it does not need to know the accurate mathematical model of a nonlinear system. Applying the proposed model and the control scheme to real systems will be our future work. The favorable performance of the WFBELC can be further verified through the test results of the hardware platform.

## Author Contributions

All authors listed have made a substantial, direct and intellectual contribution to the work, and approved it for publication.

### Conflict of Interest Statement

The authors declare that the research was conducted in the absence of any commercial or financial relationships that could be construed as a potential conflict of interest.
